# Disruption of Arterial Perivascular Drainage of Amyloid-β from the Brains of Mice Expressing the Human APOE ε4 Allele

**DOI:** 10.1371/journal.pone.0041636

**Published:** 2012-07-25

**Authors:** Cheryl A. Hawkes, Patrick M. Sullivan, Sarah Hands, Roy O. Weller, James A. R. Nicoll, Roxana O. Carare

**Affiliations:** 1 Clinical Neurosciences, Clinical and Experimental Sciences, Faculty of Medicine, University of Southampton, Southampton, United Kingdom; 2 Department of Medicine, Duke University, Durham VA Medical Center and GRECC, Durham, North Carolina, United States of America; 3 Faculty of Natural and Environmental Sciences, University of Southampton, Southampton, United Kingdom; Massachusetts General Hospital/Harvard Medical School, United States of America

## Abstract

Failure of elimination of amyloid-β (Aβ) from the brain and vasculature appears to be a key factor in the etiology of sporadic Alzheimer’s disease (AD) and cerebral amyloid angiopathy (CAA). In addition to age, possession of an apolipoprotein E (*APOE)* ε4 allele is a strong risk factor for the development of sporadic AD. The present study tested the hypothesis that possession of the *APOE* ε4 allele is associated with disruption of perivascular drainage of Aβ from the brain and with changes in cerebrovascular basement membrane protein levels. Targeted replacement (TR) mice expressing the human *APOE3* (TRE3) or *APOE4* (TRE4) genes and wildtype mice received intracerebral injections of human Aβ_40_. Aβ_40_ aggregated in peri-arterial drainage pathways in TRE4 mice, but not in TRE3 or wildtype mice. The number of Aβ deposits was significantly higher in the hippocampi of TRE4 mice than in the TRE3 mice, at both 3- and 16-months of age, suggesting that clearance of Aβ was disrupted in the brains of TRE4 mice. Immunocytochemical and Western blot analysis of vascular basement membrane proteins demonstrated significantly raised levels of collagen IV in 3-month-old TRE4 mice compared with TRE3 and wild type mice. In 16-month-old mice, collagen IV and laminin levels were unchanged between wild type and TRE3 mice, but were lower in TRE4 mice. The results of this study suggest that *APOE4* may increase the risk for AD through disruption and impedance of perivascular drainage of soluble Aβ from the brain. This effect may be mediated, in part, by changes in age-related expression of basement membrane proteins in the cerebral vasculature.

## Introduction

Accumulation of insoluble 39–42 amino acid amyloid-β (Aβ) peptides in the brain parenchyma is one of the pathological hallmarks of Alzheimer’s disease (AD). The majority of AD patients will also develop Aβ_40_ accumulation in the walls of cortical and leptomeningeal arteries as cerebral amyloid angiopathy (CAA) [1]. Increasing evidence suggests that CAA contributes to the pathophysiology of AD, as vascular Aβ deposition is associated with the death of endothelial and smooth muscle cells, pericytes as well as increased vessel tortuosity and disturbances in cerebrovascular function [1]–[4]. Clinically, CAA correlates with cerebral hypoperfusion, microhemorrhages and cognitive impairment [5]–[9].

Although age is the strongest risk factor for the development of sporadic AD, there is also a robust association between CAA, AD and possession of the apolipoprotein E (apoE) ε4 allele [10], [11]. ApoE is the predominant apolipoprotein expressed in the brain and plays an important role in the transport, uptake and redistribution of cholesterol [12], [13]. Variation in the human *APOE* gene sequence results in the presence of three alleles (ε2, ε3 and ε4), which encode the production of three corresponding protein isoforms (E2, E3 and E4). Individuals expressing one or two copies of the ε4 allele are at higher risk of developing AD, with an earlier age of onset [14]. Deposition of Aβ in capillary walls and increased severity of CAA are observed in the brains of humans and transgenic mice expressing human apoE4 [11], [15], [16]. However, the mechanisms that underlie this susceptibility are unknown.

Recent evidence supports the hypothesis that CAA is the result of incomplete clearance of Aβ from the brain [17], [18]. Multiple mechanisms mediate Aβ removal from the brain, including enzymatic degradation, uptake by microglia and macrophages, receptor-mediated transport across the endothelium and drainage within interstitial fluid (ISF) along cerebrovascular basement membranes [19], [20]. Experimental and histological evidence suggests that these clearance mechanisms are affected by expression of the apoE4 isoform. For example, levels of insulin-degrading enzyme are significantly lower in the brains of apoE4-positive transgenic mice and humans with AD, compared to those possessing apoE2 or apoE3 [21], [22]. Microglia and macrophages derived from transgenic mice expressing human apoE4 have altered cell morphology, higher production of pro-inflammatory cytokines and are less efficient at degrading Aβ than those from apoE2 and apoE3 mice [23], [24]. In addition, binding of Aβ to apoE has been shown to redirect transport across the blood-brain barrier (BBB) in an isoform specific manner (apoE4 > apoE3 or apoE2), towards slower clearance via very low density lipoprotein receptors [25]. However, little is known about the putative effect of apoE genotype on the elimination of Aβ from the brain along vascular basement membranes.

Cerebral vascular basement membranes are thin sheets of highly specialized extracellular matrix that are composed of laminins, collagen IV, nidogens and heparan sulfate proteoglycans such as perlecan and agrin [26], [27]. Basement membranes regulate endothelial and smooth muscle cell growth, differentiation and migration and are proposed to be an important pathway for the efflux of ISF from the brain [17], [26], [27]. Previous studies have demonstrated that solutes injected into the murine brain co-localize with basement membranes in the walls of capillaries and arteries [28]. Further, solute diffusion occurs rapidly and is detected in capillary walls within 5 minutes of injection and within perivascular macrophages at 30 minutes post-injection [28]. Changes in basement membrane expression and morphology are observed in the vasculature of the aging and AD brain and result in the disturbed elimination of solutes from the parenchyma and the development of CAA [3], [29], [30]. Furthermore, laminin, collagen IV, nidogen, agrin and perlecan, can interact directly with Aβ and influence its aggregation [31]–[34].

There is also evidence to suggest that apoE mediates the interaction between Aβ and basement membrane proteins. *In vitro* experiments have shown that laminin binds apoE and inhibits Aβ_40_ fibril formation induced by apoE4 [35], [36]. ApoE and Aβ co-localize in the cerebral vasculature of mouse and AD brains [37], [38] and a significant reduction in the surface area of agrin immunoreactivity has been reported previously in the brains of AD individuals homozygous for the *APOE4* allele [39]. In addition, recent findings from transgenic PDAPP mice expressing human apoE4 have demonstrated elevated levels of soluble Aβ within their cerebral ISF, without increased Aβ production [40], suggesting the possible influence of apoE on the perivascular drainage of Aβ from the brain. Therefore, in the present study, we tested the hypothesis that the presence of human apoE3 and apoE4 isoforms differentially affects the level of cerebrovascular basement membranes and the pattern of perivascular drainage of Aβ from the adult mouse brain. We found that the pattern of Aβ_40_ distribution following injection into the hippocampus was significantly disrupted in the arterial walls of 3- and 16-month old TRE4 animals, but not in TRE3 or wildtype mice. Further, analysis of vascular basement membrane protein levels showed increased collagen IV levels in 3-month old TRE4 mice compared with TRE3 and wild type mice. In 16-month-old mice, collagen IV and laminin levels were unchanged between wild type and TRE3 mice, but were lower in TRE4 mice. These results suggest that expression of apoE4 may promote Aβ accumulation in AD in part by modifying the expression of cerebral vascular basement membrane proteins and disrupting the efficiency of perivascular drainage of Aβ from the brain.

## Results

### Effect of apoE Genotype on Perivascular Drainage of Aβ

**Figure 1:**
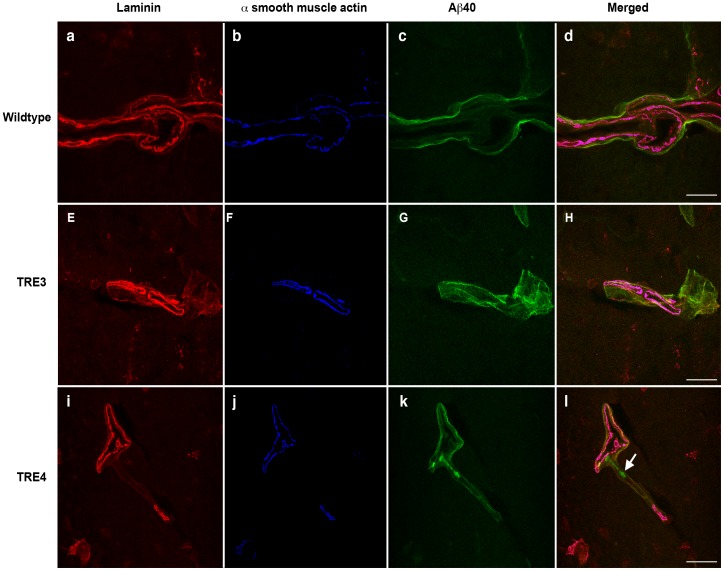
Distribution of human Aβ_40_ within the cerebral vasculature of 3-month old wildtype, TRE3 and TRE4 mice. Photomicrographs of a hippocampal leptomeningeal artery in a wildtype mouse (a–d), demonstrating the even distribution of intracerebrally-injected Aβ (green, c and d) along laminin-positive (red, a and d) basement membranes of the tunica adventitia and the tunica media (identified by the presence of α-smooth muscle actin, blue, b and d). Co-localization of laminin, α-smooth muscle actin and Aβ is observed as bright pink. In the TRE3 mice (e–h), Aβ (green, g and h) also distributed evenly along the entire length of laminin-positive (red, e and h) basement membranes in the tunica media of a hippocampal artery (blue, f and h). By contrast, in the TRE4 mice (i–l), Aβ (green, k and l) was uniformly distributed along the basement membrane of the glia limitans (red, i and l), but appeared aggregated (arrow) in the basement membrane within the tunica media of the hippocampal artery (blue, j and l). Scale bars: a–d = 10 µm; e–h = 25 µm; i–l = 25 µm.

**Figure 2:**
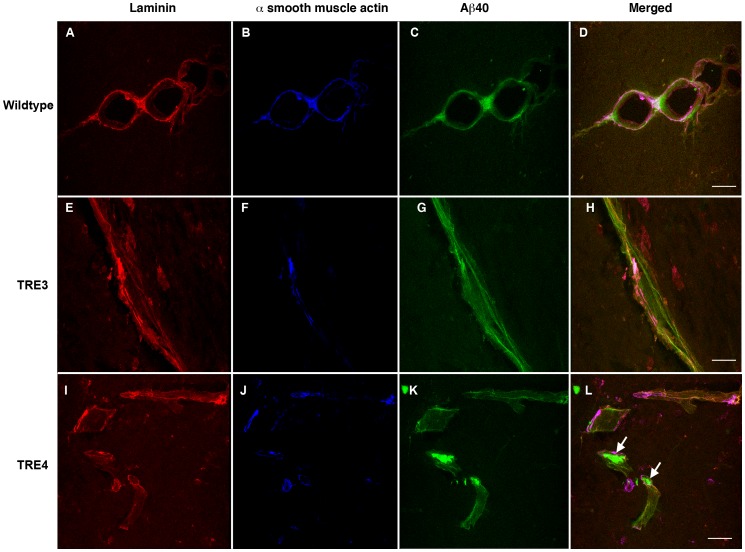
Distribution of human Aβ_40_ within the cerebrovasculature of 16-month old wildtype, TRE3 and TRE4 mice. Photomicrographs of a transverse section of a bifurcating leptomeningeal artery in a wildtype mouse (a–d), demonstrating the distribution of intracerebrally-injected Aβ (green, c and d) along laminin-positive (red, a and d) basement membranes in the tunica media (identified by the presence of α-smooth muscle actin, blue, b and d). Co-localization of laminin, α-smooth muscle actin and Aβ is observed as white or bright pink. In the TRE3 mice (e–h), Aβ (green, g and h) distributed along the entire length of laminin-positive (red, e and h) basement membranes of the tunica adventitia and in the tunica media of a hippocampal leptomeningeal artery (blue, f and h). In the TRE4 mice (i–l), most of the Aβ (green, k and l) was observed as large deposits (arrows) in the basement membrane (red, i and l) of hippocampal arteries (blue, j and l). Scale bars  = 25 µm.

To evaluate the effect of apoE isoform on perivascular clearance of Aβ from the parenchyma, 3- and 16-month old wildtype, TRE3 and TRE4 mice were injected into the left hippocampus with non-fibrillar, oligomeric HiLyte Fluor™ 488-labeled human Aβ_40_ (Fig. S1a and b). Fluorescently-labeled Aβ peptides have been used to assess Aβ uptake into neurons, microglia and transport across the BBB, suggesting that fluorophore attachment does not alter the ability of clearance mechanisms to interact with and remove Aβ from the brain [41]–[43]. Aβ distribution was evaluated following a ten-minute diffusion period to allow for adequate perivascular drainage while minimizing Aβ clearance across the BBB or uptake by perivascular macrophages, as previously demonstrated [25], [28]. Similar to the distribution of Aβ in human CAA [17], intracerebrally-injected Aβ co-localized with laminin-positive basement membranes in the tunica adventitia and the tunica media of leptomeningeal arteries and in the glia limitans and tunica media of the hippocampal arteries in wildtype and apoE TR mice (Figs. 1 and 2). In 3-month old wildtype mice, Aβ distributed in a thin, even layer along the entire length of the vessel wall (Fig. 1a–d). A similar pattern of distribution was noted in the brains of young TRE3 mice (Fig. 1e–h). The profile of Aβ labeling in young TRE4 mice was mixed, with Aβ evenly distributed along some portions of the blood vessels, and with a patchy, amorphous appearance in other regions (Fig. 1i–l). In the 16-month old animals, the distribution of Aβ along the vessels of wildtype mice resembled that in the young mice, albeit with a rougher, thicker appearance, which corresponded to age-related changes in laminin morphology (Fig. 2a–d). Likewise, the appearance of Aβ in the aged TRE3 mice was evenly distributed along the length of the vessel wall (Fig. 2e–h). By contrast, in the 16-month old TRE4 mice, most of the Aβ was observed as large deposits that appeared to have aggregated in the vessel walls (Fig. 2i–l). These Aβ aggregates were identified in vessels throughout the hippocampus at varying distances from the site of injection, suggesting that their deposition was not due to fibrillization upon injection. The number of Aβ deposits was significantly higher in the hippocampi of TRE4 mice than in the TRE3 mice, at both 3- and 16-months of age (p<0.05, Table 1). No differences were noted in hippocampal blood vessel density between TRE3 and TRE4 mice at any age (data not shown).

**Table 1:** Number of Aβ deposits in the hippocampi of 3- and 16-month old TRE3 and TRE4 mice.

	3 months	16 months
**TRE3**	1.5±0.3	1.2±0.8
**TRE4**	2.7±0.9*	3.9±0.6*

* p<0.05, TRE3 vs. TRE4.

To determine if the pattern of Aβ deposition in the TRE4 mice was due to binding of human Aβ_40_ to vascular deposits of endogenous mouse Aβ [44], brain tissue sections from aged TRE4 mice were processed for Aβ immunoreactivity using an anti-Aβ antibody (clone 4G8) that recognized epitopes of both mouse and human Aβ. The anti-Aβ antibody localized predominantly to the human Aβ that had been injected into the ipsilateral hippocampus (Fig. S1c and d), suggesting that the Aβ aggregates were not precipitated by previously seeded Aβ.

**Figure 3:**
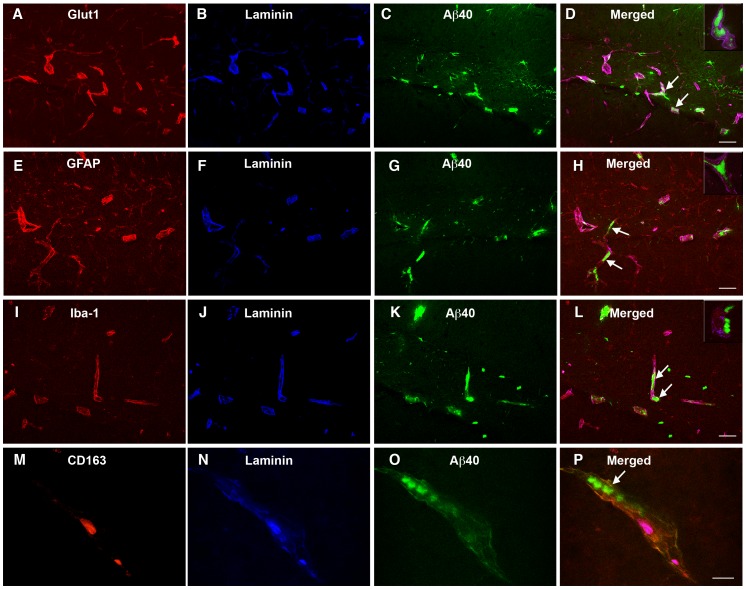
Localization of the Aβ deposits in the blood vessels of 16-month old TRE4 mice. Double labeling immunocytochemistry was used to assess the location of Aβ (green) aggregates (arrows) with respect to laminin-positive basement membranes (blue) in hippocampal arteries and glut-1-positive endothelial cells (red, a–d), GFAP-positive astrocytes (red, e–h), Iba-1-positive juxtavascular microglia (red, i–l) and CD163-positive perivascular macrophages (red, m–p). No co-localization was observed between Aβ aggregates and any cell type, suggesting that the Aβ deposits do not represent intracellular aggregates. Inserts represent x63 magnification. Scale bar: a–1 = 50 µm; m–p = 25 µm.

To assess the localization of the vascular Aβ deposits in the aged TRE4 mice in more detail, triple labeling immunocytochemistry was performed using an anti-laminin antibody in combination with antibodies against α-smooth muscle actin, glut-1, GFAP, Iba-1 or CD163. No co-localization was noted between the Aβ aggregates and either smooth muscle cells (Fig. 2i–l), endothelial cells (Fig. 3a–d), astrocytes (Fig. 3e–h), juxtavascular microglia (Fig. 3i–l) or perivascular macrophages (Fig. 3m–p), indicating that the observed Aβ deposits do not represent intracellular aggregates, but are localized to the cerebrovascular basement membrane. This is further supported by the strong similarity between the pattern of Aβ deposition (Fig. 2l and Fig. 3p) and that observed for laminin staining in TRE4 mice (Fig. S1e and f). Staining for mouse IgG was negative in wildtype, TRE3 and TRE4 mice of all ages and was not detected in Aβ deposits (data not shown), suggesting that BBB integrity was not generally compromised or responsible for Aβ accumulation in the TRE4 mice. In addition, double labeling of tissue sections from aged TRE3 and TRE4 mice for laminin and apoE, demonstrated co-localization of apoE with the basement membranes in blood vessels in both TRE3 and TRE4 mice (Fig. S1g and h). The degree of apoE staining appeared higher in arteries of TRE4 mice, but did not co-localize significantly with the Aβ deposits observed in these animals (Fig. S1g and h).

### Cerebrovascular Basement Membranes in Young and Old TRE3 and TRE4 Mice

**Figure 4:**
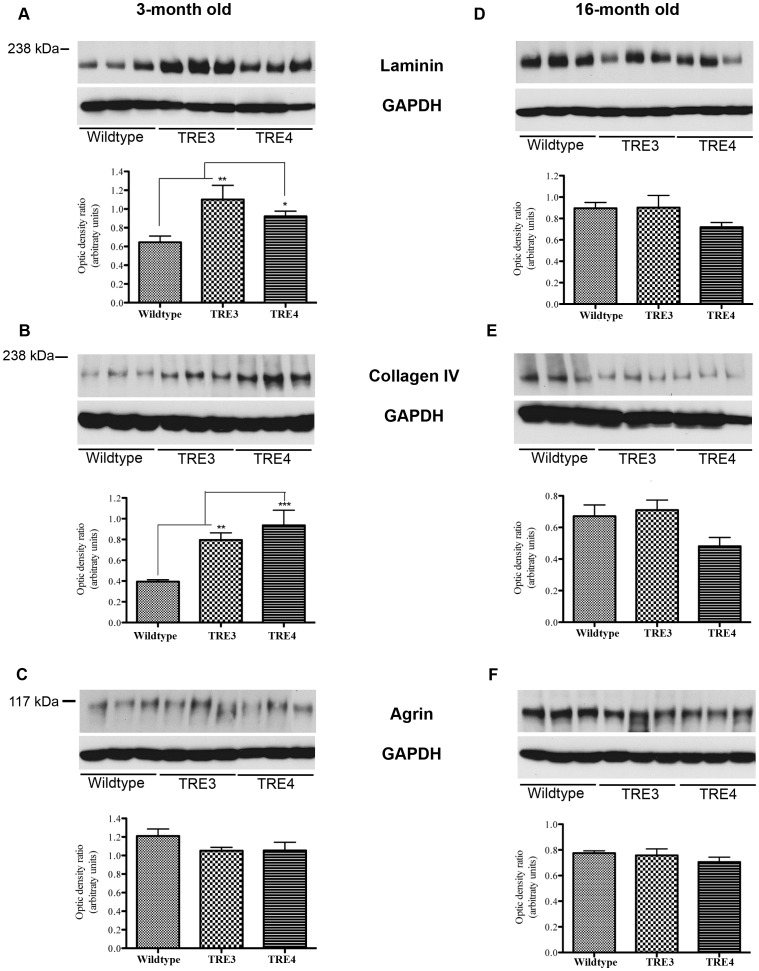
Basement membrane protein levels in the brains of 3- and 16-month old wildtype, TRE3 and TRE4 mice. Frontal-parietal brain homogenates in 3-month old (a–c) and 16-month-old (d–f) TRE3, TRE4 and wildtype controls were analyzed by Western blotting for the levels of laminin (a, d), collagen IV (b, e) and agrin (c, f). Levels of laminin and collagen IV were significantly higher in the brains of 3-month old, but not 16-month old TRE3 and TRE4 mice versus wildtype mice. Agrin levels were unchanged between wildtype, TRE3 and TRE4 mice at either age. Optic density ratios of protein to GAPDH levels from 3 blots per antibody were analyzed by repeated measures two-way ANOVA with Bonferroni post-hoc test. Histograms represent mean optic density ratio values ± S.E.M., *p<0.05, **p<0.01, ***p<0.001.

We have previously found that alterations in perivascular clearance of solutes from the brain are associated with changes in basement membrane levels [30]. To determine if apoE genotype differentially affects basement membrane protein levels, fronto-parietal cortices from 3- and 16-month old wildtype, TRE3 and TRE4 mice were assessed for laminin, collagen IV and agrin levels by Western blotting. In the 3-month old mice, the levels of laminin were significantly higher in TRE3 and TRE4 mice compared to aged-matched wildtype mice (Fig. 4a, p<0.01 and p<0.05, respectively). Significantly more collagen IV was also noted in young TRE3 and TRE4 mice versus wildtype controls (Fig. 4b, p<0.01 and p<0.001, respectively). Agrin levels were unaltered between young wildtype, TRE3 and TRE4 mice (Fig. 4c).

Analysis of brain homogenates from 16-month old animals did not reveal significant differences in the level of laminin between wildtype, TRE3 and TRE4 mice, although there was a trend towards decreased laminin in the TRE4 mice (Fig. 4d). Similarly, there was a trend towards decreased levels of collagen IV in the TRE4 animals (Fig. 4e). No significant differences were noted in the amount of agrin (Fig. 4f) in the brains of 16-month old wildtype, TRE3 and TRE4 mice. To evaluate the effect of *APOE* genotype on changes in basement membrane levels with age, the ratios of laminin and collagen IV between 3- and 16-months of age were calculated (Table 2). An age-dependent decrease in laminin was noted for both TRE3 and TRE4 mice, with no significant difference between ratios (TRE3 = 0.82±0.11 vs. TRE4 = 0.78±0.5, p>0.05). Collagen IV levels also decreased as a function of age in TRE3 and TRE4 mice, but this decrease was significantly larger in the TRE4 mice (TRE3 = 0.89±0.08 vs. TRE4 = 0.51±0.07; p<0.05).

**Table 2:** Percent change in basement membrane levels of TRE3 and TRE4 mice with age.

	Laminin	Collagen IV	Agrin
**TRE3**	−18.1±13.2	−10.8±8.7	+28.1±6.6
**TRE4**	−22.1±6.1	−48.6±13.6*	+33.1±14.0

* p<0.05, TRE3 vs. TRE4.

**Figure 5:**
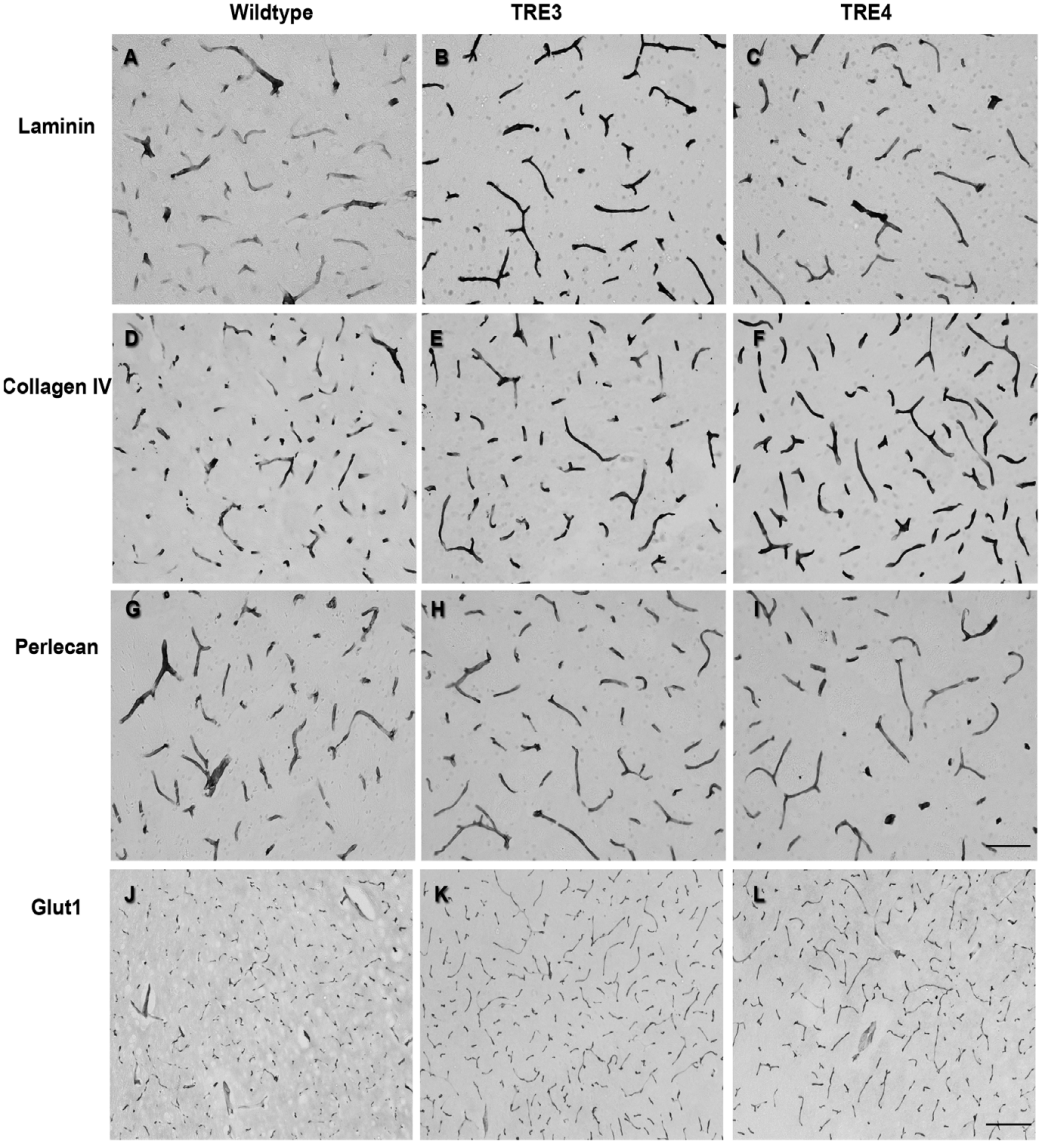
Levels and morphology of cerebrovascular basement membrane proteins in cortical capillaries of 3-month old wildtype, TRE3 and TRE4 mice. Brain tissue sections from wildtype (a, d, g, j), TRE3 (b, e, h, k) and TRE4 mice (c, f, i, l) were processed for laminin, collagen IV and perlecan immunocytochemistry. The staining intensity of both laminin (a–c) and collagen IV (d–f) was higher in TRE3 and TRE4 mice, compared to wildtype animals. Perlecan levels were constant across genotypes (g–i). No differences were noted in the pattern of glut-l labeling of endothelial cells between mouse groups (j–l). Scale bars: a–l = 100 µm; m–o = 200 µm.

To determine if the observed differences in basement membrane proteins were focal or generalized, brain tissue sections from 3- and 16-month old wildtype, TRE3 and TRE4 mice were assessed by immunocytochemistry for laminin and collagen IV. Due to technical difficulties in achieving adequate antigen retrieval for agrin immunocytochemistry, an anti-perlecan antibody was used as a marker of heparan sulfate proteoglycans. Consistent with the Western blot data, increased staining intensity for laminin in the basement membranes of capillaries was noted in both 3-month old TRE3 and TRE4 mice compared to age-matched wildtype mice (Fig. 5a–c). More intense labeling of collagen IV was also observed in the capillaries of young TRE4 mice than in the TRE3 and wildype mice (Fig. 5d–f). No significant differences were noted in the intensity of perlecan immunoreactivity of capillary basement membranes between wildtype, TRE3 or TRE4 mice (Fig. 5g–i). The pattern of expression of glut-l as a general marker for endothelial cells was comparable between all groups of 3-month old mice (Fig. 5j–l), suggesting that the observed differences in laminin and collagen IV levels were not due to changes in capillary density.

**Figure 6:**
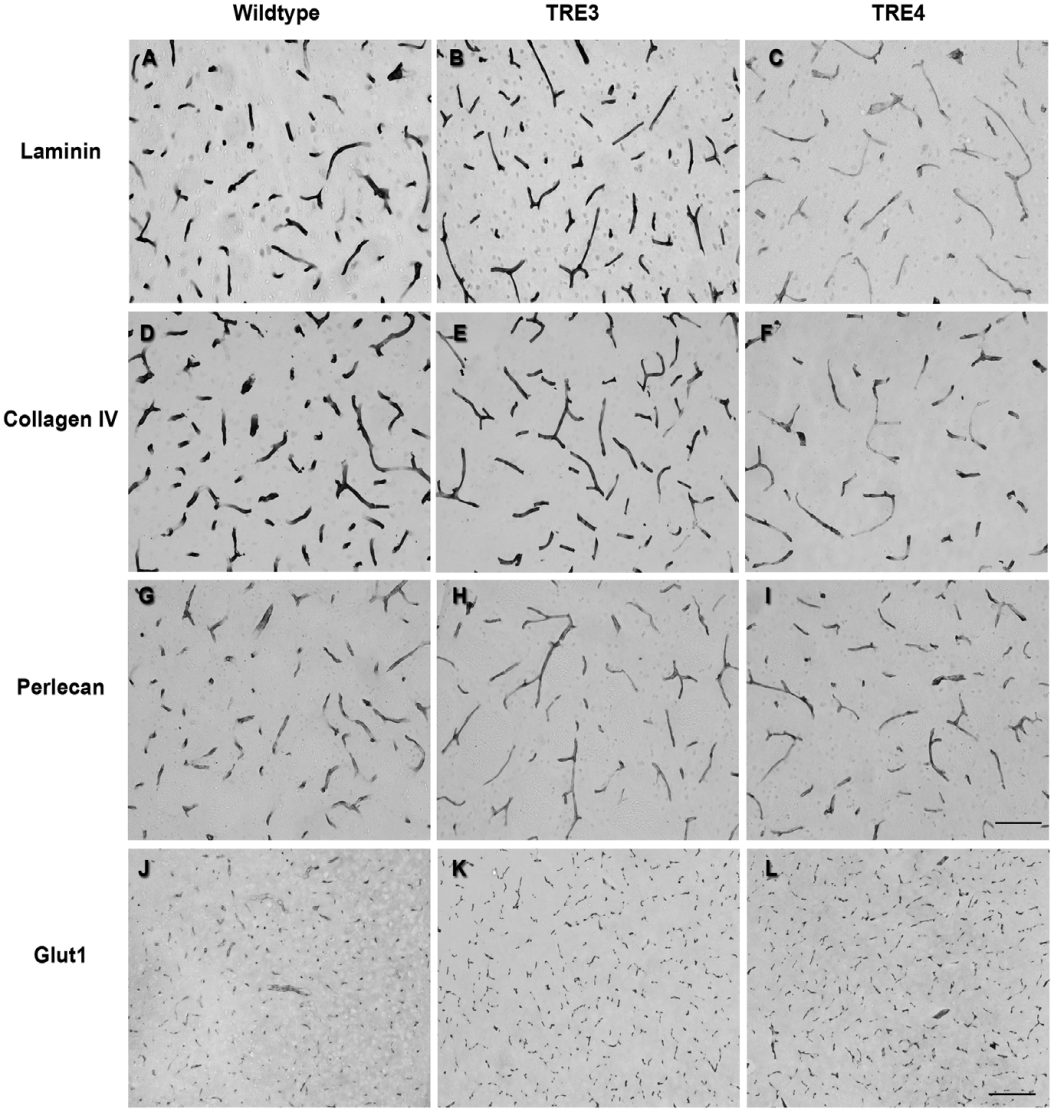
Cerebrovascular basement membrane levels and morphology in cortical capillaries of 16-month old wildtype, TRE3 and TRE4 mice. Brain tissue sections from wildtype (a, d, g, j), TRE3 (b, e, h, k) and TRE4 mice (c, f, i, l) were processed by immunocytochemistry for laminin, collagen IV and perlecan. The staining intensity of laminin (a–c) and collagen IV (d–f) was significantly lower in the blood vessels of TRE4 mice and had a patchy appearance compared to wildtype and TRE3 mice. Perlecan levels remained unchanged across genotypes (g–i), as did the pattern of glut-l endothelial cell labeling (j–l). Scale bars: a–l = 100 µm; m–o = 200 µm.

In capillaries of 16-month old mice, the intensity of laminin immunolabeling was comparable between aged wildtype and TRE3 mice, but was significantly decreased in the brains of TRE4 mice (Fig. 6a–c). The staining pattern of collagen IV in the capillaries of TRE4 mice was patchy, with large portions of the vessels showing significantly lower intensity of staining, compared to both TRE3 and wildtype mice (Fig. 6d–f). No differences were noted in the staining intensity or pattern of perlecan labeling between 16-month-old wildtype, TRE3 and TRE4 mice (Fig. 6g–i). No variance was noted in glut-1 immunolabeling between mouse genotypes at this age (Fig. 6j–l).

## Discussion

Possession of the *APOE4* allele is one of the strongest risk factors for the development of sporadic AD and CAA, but the exact mechanisms that underlie this susceptibility are unknown. We tested the hypothesis that perivascular drainage of Aβ from the brain is disrupted in the presence of apoE4, in association with changes in cerebrovascular basement membrane levels. We found that Aβ_40_ injected into the hippocampi of TRE4 mice accumulated within the basement membranes of blood vessels, beginning at 3 months of age and became prominent at 16 months. In addition, levels of laminin and collagen IV were higher in the brains of TRE4 mice at 3 months of age, but lower than those of wildtype and TRE3 mice by 16 months of age. These results suggest that possession of apoE4 changes the levels of basement membrane proteins in cerebral blood vessels and disrupts the efficiency of perivascular drainage of Aβ from the brain.

A role for apoE4 in the onset and severity of CAA has previously been suggested in the brains of transgenic mice expressing human apoE [11], [15]. Fryer et al. [15] showed that breeding of Tg2576 transgenic mice onto a human *APOE3/3* background delayed the development of Aβ plaques in the parenchyma and prevented the development of CAA, while those on an *APOE4/4* background had delayed Aβ deposition but more extensive CAA. Recently, Castellano et al. [40] demonstrated that the half-life and cerebral levels of Aβ in the ISF were increased in both young and old TgPDAPP mice possessing human apoE4, compared to those expressing apoE2 and apoE3. Similarly, we found that perivascular drainage of Aβ contained within hippocampal ISF was disrupted in the brains of 3- and 16-month old TRE4 mice. These data suggest that the increase in CAA severity observed in mice and humans possessing apoE4 may be due in part to its inefficient drainage along cerebrovascular basement membranes. Moreover, the presence of Aβ deposits in the vasculature of 3-month old TRE4 mice, suggests that the disruption of perivascular drainage of Aβ from the brain occurs early and is in line with the pre-symptomatic effects of apoE4 on Aβ concentrations, white matter structure and cognition that have been reported in young transgenic mice and humans [40], [45], [46].

The mechanisms that underlie the accumulation of vascular Aβ have not been fully elucidated. Aβ is degraded by enzymes, such as neprilysin and insulin-degrading enzyme, and removed from the brain by uptake by microglia and macrophages and via lipoprotein receptor-related protein 1 (LRP)-mediated transport across the endothelium [19], [21]–[24], [47]–[50]. Recent work has demonstrated an important role for serum response factor and myocardin in regulating LRP-1 levels in cerebral vascular smooth muscle cells, which are also involved in Aβ clearance [51]. In addition, Aβ secreted into the cerebral interstitial fluid is removed from the brain by bulk flow along the basement membranes of capillary and artery walls [17], [28], [30]. Changes in cerebrovascular basement membranes, including thickening, reduplication and vacuolization are observed in the aging brain and may contribute to the etiology of AD and CAA [3], [29]. Further, aggregation of Aβ has been shown to be inhibited in the presence of laminin, collagen IV and nidogen but accelerated by agrin and perlecan [31]–[34]. In the present study, we found that the levels of laminin and collagen IV were differentially altered between 3- and 16-month old wildtype, TRE3 and TRE4 mice, while levels of agrin and perlecan were unchanged. These results suggest that i) replacement of the mouse apoE gene with the human homolog affects basement membrane synthesis and/or degradation in the mouse brain and ii) that APOE genotype regulates the levels of basement membrane proteins. Moreover, when analyzed across the lifespan, the change in the levels of collagen IV between 3- and 16-months of age appeared greater in TRE4 mice compared to TRE3 animals, suggesting that apoE genotype might also influence the degree to which basement membrane expression changes during aging.

Previous reports in human AD and TgAPP mice have suggested a localization of apoE in blood vessels in the brain [37], [38], but it is not clear if apoE specifically associates with the basement membrane or mediates the perivascular clearance of Aβ. We found that apoE co-localized with laminin in the blood vessels of both TRE3 and TRE4 mice. Although TRE4 mice express endogenously lower hippocampal levels of apoE than TRE3 mice [52], the apoE staining appeared more robust in the vessels of aged TRE4 vs. TRE3 mice. However, we did not observe a significant overlap between apoE and deposits of Aβ in the vessels of either TRE3 or TRE4 mice. The present study suggests that impaired Aβ clearance from the brains of TRE4 mice is due predominantly to alterations in basement membrane proteins. In light of the increasing importance that apoE genotype appears to play on the efficacy of AD therapeutics [53] and the vascular related side effects of therapeutic removal of Aβ [54], these data highlight the importance of addressing apoE-associated changes in the aging cerebrovasculature in the design and interpretation of new therapeutics for the treatment strategies of AD and CAA.

## Materials and Methods

### Animals

Homozygous, male TRE3 and TRE4 mice [55] were maintained on a C57BL/6 background and allowed food and water *ad libitum*. Experiments were performed using 3–4 and 16–17 month-old TRE3, TRE4 mice and age-matched C57BL/6 controls. All experiments were performed in accordance with animal care guidelines stipulated by the Animal Care and Use Committee at the University of Southampton. The protocol was approved by the Home Office under the Animals Scientific Procedures Act 1986 (project license 30/2008) and all efforts were made to minimize pain and suffering.

### Aβ Peptide Preparation and Atomic Force Microscopy (AFM)

HiLyte Fluor™ 488-labeled human Aβ_40_ (100 mg, Cambridge Biosciences, UK) was solubilized in 50 µL 1% NH_4_OH, vortexed for 30 seconds and made up to 100 µM with ice-cold sterile PBS. To determine the aggregation of status of Aβ, freshly prepared Aβ or aliquots incubated in PBS for 24 hours at 37°C were evaluated by AFM. Aβ (5 µL) was spotted onto a freshly cleaved mica disc (Agar Scientific, Stansted, UK) and imaged in air with a digital multimode Nanoscope III AFM (Veeco, Mannheim, Germany) operating in tapping mode with uncoated silicon cantilevers (FM-W, Nanoworld Innovative Technologies, Switzerland, nominal spring constant 2.8 N/m).

### Intracerebral Injections

3- and 16-month old wildtype and apoE TR mice (n = 4/group) were injected stereotaxically with 0.5 µL ice-cold Aβ_40_ into the left hippocampus (coordinates from Bregma: AP = −2 mm; ML = 1.8 mm and DV = 1.6 mm). Injection capillaries were left *in situ* for 2 minutes to prevent reflux along the capillary and mice were sacrificed 10 minutes post-injection to allow for adequate perivascular drainage while minimizing Aβ clearance across the BBB or via uptake by microglia or macrophages [25], [28]. Mice were intracardially perfused with phosphate buffered saline (PBS, pH 7.4), followed by 4% paraformaldehyde and brains processed for double labeling immunocytochemistry.

### Double Labeling Immunocytochemistry

Brain sections (20 µm thickness) were incubated overnight with anti-laminin (1∶500, Sigma-Aldrich, Dorset, UK), developed with AlexaFluor 546-conjugated anti-rabbit (1∶200; Invitrogen, Paisley, UK) and then incubated with anti-α smooth muscle actin (1∶500; Sigma-Aldrich), anti-glucose transporter-1 (glut-1, Sigma-Aldrich, 1∶500), anti-glial fibrillary acidic protein (GFAP, 1∶1000; Dako, Ely, UK) or anti-apoE (polyclonal against human apoE, 1∶250; Merck, Nottingham, UK). The next day, sections were rinsed in PBS and developed with the appropriate fluorescently conjugated secondary antibody (1∶200). Antigen retrieval with formic acid or citrate buffer was performed for double labeling with anti-Aβ_17−24_ (clone 4G8, 1∶100, Cambridge Biosciences, Cambridge, UK), anti-Iba-1 (1∶500; Wako Chemicals, Eastleigh, UK) or anti-CD163 antibodies (1∶200; AbD Serotec, Kidlington, UK). Photomicrographs were captured using a Leica SP5 confocal laser scanning microscope (Milton Keys, UK) and exported to Photoshop CS software.

### Western Blotting

Wildtype and apoE TR mice (n = 4/group) were perfused intracardially with PBS. Brain tissues were sonicated in Ripa lysis buffer [20 mM Tris-HCl (pH 8.0), 150 mM NaCl, 1 mM EDTA, 0.1% SDS, 1% Igepal, 50 mM NaF, 1 mM NaVO_3_] containing a protease inhibitor cocktail (Merck, Nottingham, UK) and supernatants (15–30 µg) were separated by gel electrophoresis on 3–8% Tris-acetate gels (Invitrogen, Paisley, UK) and transferred onto a nitrocellulose membrane. Collagen IV samples were prepared without β-mercaptoethanol or heat. Membranes were incubated overnight at 4°C with anti-collagen IV **(**1∶500; Abcam, Cambridge, UK), anti-laminin (1∶500, Sigma-Aldrich, Dorset, UK) or anti-agrin (1∶750; Millipore, Watford, UK**)** antibodies. Membranes were stripped and reproved with anti-glyceraldehyde-3-PDH (GAPDH) antibody (1∶50,000; Sigma-Aldrich) to ensure equal protein loading. Immunoblots were quantified by densitometry using Image J software (NIH, Maryland, USA) and calculated as an optical density ratio of protein levels normalized to GAPDH levels.

### Enzyme-linked Immunocytochemistry

Wildtype and apoE TR mice (n = 4/group) mice were perfused with 0.1 M PBS followed by 4% paraformaldehyde. Tissue sections (20 µm thickness) were treated for 2 minutes at 37°C with pepsin from porcine gastric mucosa (1 mg/mL in 0.2N HCl, Sigma-Aldrich) and incubated overnight with anti-laminin (1∶500), anti-collagen IV **(**1∶500), anti-perlecan (1∶500; Millipore, Watford, UK) or anti-glut-1 **(**1∶500) antibodies. Sections were washed with PBS, incubated with anti-rat or anti-rabbit horseradish peroxidase conjugates (1∶400; Vector Labs, Peterborough, UK) and developed with nickel-enhanced diaminobenzidine as chromogen.

### Statistical Analysis

Assessment of perivascular drainage and basement membrane levels was carried out in the hippocampus and fronto-parietal cortex, respectively, as representative brain areas that are most susceptible to the development to murine CAA. Histograms of western blots were compiled from 3 blots using mean ± S.E.M. values and analyzed using repeated measures one-way ANOVA with Newman-Keuls post-hoc test or one-way Student’s t-test (significance set at p<0.05). The number of hippocampal blood vessels containing Aβ deposits in a 775 µm×775 µm field (4 fields/mouse) were counted manually and analyzed using a Student’s t-test (significance set at p<0.05). For quantification of hippocampal vessel density, micrographs of glut-1 staining were converted to binary images (4 sections/mouse), evaluated by densitometry using Image J software and evaluated using a one-way ANOVA with Newman-Keuls post-hoc test (significance p<0.05).

## Supporting Information

Figure S1 **Soluble Aß injected into the hippocampus of TRE4 mice drains along basement membranes and co-localizes with apoE.** a and b: HiLyte Fluor™ 488-labeled human Aβ_40_ used for intra-hippocampal injections was confirmed by atomic force microscopy to be oligomeric (a), compared to the fibrillar Aβ that resulted following 24 hrs incubation at 37°C (b). c and d: Brain tissue sections from 16-month old TRE4 mice were processed for Aβ immunoreactivity using a pan anti-Aβ antibody that recognized both mouse and human Aβ. The anti-Aβ antibody localized predominantly to the human Aβ (green) that had been injected into the ipsilateral hippocampus, while little to no mouse Aβ (red) was detected in the vessels in either the ipsi- (c) or contralateral hippocampus (d). e and f: Laminin staining in the vessel wall of capillaries in the 16-month old TRE4 mice (arrows) matched that of the Aβ deposits in the same animals. g and h: Double labeling immunocytochemistry with antibodies against laminin (blue) and apoE (red) in 16-month old TRE3 (g) and TRE4 (h) mice injected with human Aβ_40_ (green), showed localization of apoE with the basement membrane in cortical and leptomeningeal arteries. Scale bars: a and b = 10 µm; c and d = 50 µm; e–h = 25 µm.
(TIF)

## References

[1] Haglund M, Kalaria R, Slade JY, Englund E (2006). Differential deposition of amyloid beta peptides in cerebral amyloid angiopathy associated with Alzheimer’s disease and vascular dementia.. Acta Neuropathol.

[2] Miao J, Xu F, Davis J, Otte-Holler I, Verbeek MM (2005). Cerebral microvascular amyloid beta protein deposition induces vascular degeneration and neuroinflammation in transgenic mice expressing human vasculotropic mutant amyloid beta precursor protein.. Am J Pathol.

[3] Perlmutter LS (1994). Microvascular pathology and vascular basement membrane components in Alzheimer’s disease.. Mol Neurobiol.

[4] Tian J, Shi J, Smallman R, Iwatsubo T, Mann DM (2006). Relationships in Alzheimer’s disease between the extent of Abeta deposition in cerebral blood vessel walls, as cerebral amyloid angiopathy, and the amount of cerebrovascular smooth muscle cells and collagen.. Neuropathol Appl Neurobiol.

[5] Natte R, Maat-Schieman ML, Haan J, Bornebroek M, Roos RA (2001). Dementia in hereditary cerebral hemorrhage with amyloidosis-Dutch type is associated with cerebral amyloid angiopathy but is independent of plaques and neurofibrillary tangles.. Ann Neurol.

[6] Pfeifer LA, White LR, Ross GW, Petrovitch H, Launer LJ (2002). Cerebral amyloid angiopathy and cognitive function: the HAAS autopsy study.. Neurology.

[7] Pfeifer M, Boncristiano S, Bondolfi L, Stalder A, Deller T (2002). Cerebral hemorrhage after passive anti-Abeta immunotherapy.. Science.

[8] Chung YA, O JH, Kim JY, Kim KJ, Ahn KJ (2009). Hypoperfusion and ischemia in cerebral amyloid angiopathy documented by 99mTc-ECD brain perfusion SPECT.. J Nucl Med.

[9] Shin HK, Jones PB, Garcia-Alloza M, Borrelli L, Greenberg SM (2007). Age-dependent cerebrovascular dysfunction in a transgenic mouse model of cerebral amyloid angiopathy.. Brain.

[10] Farrer LA, Cupples LA, Haines JL, Hyman B, Kukull WA (1997). Effects of age, sex, and ethnicity on the association between apolipoprotein E genotype and Alzheimer disease. A meta-analysis. APOE and Alzheimer Disease Meta Analysis Consortium.. JAMA.

[11] Premkumar DR, Cohen DL, Hedera P, Friedland RP, Kalaria RN (1996). Apolipoprotein E-epsilon4 alleles in cerebral amyloid angiopathy and cerebrovascular pathology associated with Alzheimer’s disease.. Am J Pathol.

[12] Mahley RW, Innerarity TL (1983). Lipoprotein receptors and cholesterol homeostasis.. Biochim Biophys Acta.

[13] Masliah E, Mallory M, Veinbergs I, Miller A, Samuel W (1996). Alterations in apolipoprotein E expression during aging and neurodegeneration.. Prog Neurobiol.

[14] Blacker D, Haines JL, Rodes L, Terwedow H, Go RC (1997). ApoE-4 and age at onset of Alzheimer’s disease: the NIMH genetics initiative.. Neurology.

[15] Fryer JD, Simmons K, Parsadanian M, Bales KR, Paul SM (2005). Human apolipoprotein E4 alters the amyloid-beta 40:42 ratio and promotes the formation of cerebral amyloid angiopathy in an amyloid precursor protein transgenic model.. J Neurosci.

[16] Thal DR, Papassotiropoulos A, Saido TC, Griffin WS, Mrak RE (2010). Capillary cerebral amyloid angiopathy identifies a distinct APOE epsilon4-associated subtype of sporadic Alzheimer’s disease.. Acta Neuropathol.

[17] Weller RO, Subash M, Preston SD, Mazanti I, Carare RO (2008). Perivascular drainage of amyloid-beta peptides from the brain and its failure in cerebral amyloid angiopathy and Alzheimer’s disease.. Brain Pathol.

[18] Mawuenyega KG, Sigurdson W, Ovod V, Munsell L, Kasten T (2010). Decreased Clearance of CNS β-Amyloid in Alzheimer’s Disease.. Science.

[19] Bell RD, Zlokovic BV (2009). Neurovascular mechanisms and blood-brain barrier disorder in Alzheimer’s disease.. Acta Neuropathol.

[20] Weller RO, Cohen NR, Nicoll JA (2004). Cerebrovascular disease and the pathophysiology of Alzheimer’s disease. Implications for therapy.. Panminerva Med.

[21] Cook DG, Leverenz JB, McMillan PJ, Kulstad JJ, Ericksen S (2003). Reduced hippocampal insulin-degrading enzyme in late-onset Alzheimer’s disease is associated with the apolipoprotein E-epsilon4 allele.. Am J Pathol.

[22] Du J, Chang J, Guo S, Zhang Q, Wang Z (2009). ApoE 4 reduces the expression of Abeta degrading enzyme IDE by activating the NMDA receptor in hippocampal neurons.. Neurosci Lett.

[23] Vitek MP, Brown CM, Colton CA (2009). APOE genotype-specific differences in the innate immune response.. Neurobiol Aging.

[24] Zhao L, Lin S, Bales KR, Gelfanova V, Koger D (2009). Macrophage-mediated degradation of beta-amyloid via an apolipoprotein E isoform-dependent mechanism.. J Neurosci.

[25] Deane R, Sagare A, Hamm K, Parisi M, Lane S (2008). apoE isoform-specific disruption of amyloid beta peptide clearance from mouse brain.. J Clin Invest.

[26] Couchman JR, Abrahamson DR, McCarthy KJ (1993). Basement membrane proteoglycans and development.. Kidney Int.

[27] Timpl R (1996). Macromolecular organization of basement membranes.. Curr Opin Cell Biol.

[28] Carare RO, Bernardes-Silva M, Newman TA, Page AM, Nicoll JA (2008). Solutes, but not cells, drain from the brain parenchyma along basement membranes of capillaries and arteries: significance for cerebral amyloid angiopathy and neuroimmunology.. Neuropathol Appl Neurobiol.

[29] van Horssen J, Otte-Holler I, David G, Maat-Schieman ML, van den Heuvel LP (2001). Heparan sulfate proteoglycan expression in cerebrovascular amyloid beta deposits in Alzheimer’s disease and hereditary cerebral hemorrhage with amyloidosis (Dutch) brains.. Acta Neuropathol.

[30] Hawkes CA, Hartig W, Kacza J, Schliebs R, Weller RO (2011). Perivascular drainage of solutes is impaired in the ageing mouse brain and in the presence of cerebral amyloid angiopathy.. Acta Neuropathol.

[31] Cotman SL, Halfter W, Cole GJ (2000). Agrin binds to beta-amyloid (Abeta), accelerates abeta fibril formation, and is localized to Abeta deposits in Alzheimer’s disease brain.. Mol Cell Neurosci.

[32] Castillo GM, Ngo C, Cummings J, Wight TN, Snow AD (1997). Perlecan binds to the beta-amyloid proteins (A beta) of Alzheimer’s disease, accelerates A beta fibril formation, and maintains A beta fibril stability.. J Neurochem.

[33] Kiuchi Y, Isobe Y, Fukushima K, Kimura M (2002). Disassembly of amyloid beta-protein fibril by basement membrane components.. Life Sci.

[34] Bronfman FC, Alvarez A, Morgan C, Inestrosa NC (1998). Laminin blocks the assembly of wild-type A beta and the Dutch variant peptide into Alzheimer’s fibrils.. Amyloid.

[35] Huang DY, Weisgraber KH, Strittmatter WJ, Matthew WD (1995). Interaction of apolipoprotein E with laminin increases neuronal adhesion and alters neurite morphology.. Exp Neurol.

[36] Monji A, Tashiro K, Yoshida I, Hayashi Y, Tashiro N (1998). Laminin inhibits A beta 40 fibril formation promoted by apolipoprotein E4 in vitro.. Brain Res.

[37] Rolyan H, Feike AC, Upadhaya AR, Waha A, Van Dooren T (2011). Amyloid-beta protein modulates the perivascular clearance of neuronal apolipoprotein E in mouse models of Alzheimer’s disease.. J Neural Transm.

[38] Thal DR, Larionov S, Abramowski D, Wiederhold KH, Van Dooren T (2007). Occurrence and co-localization of amyloid beta-protein and apolipoprotein E in perivascular drainage channels of wild-type and APP-transgenic mice.. Neurobiol Aging.

[39] Salloway S, Gur T, Berzin T, Tavares R, Zipser B (2002). Effect of APOE genotype on microvascular basement membrane in Alzheimer’s disease.. J Neurol Sci.

[40] Castellano JM, Kim J, Stewart FR, Jiang H, Demattos RB (2011). Human apoE Isoforms Differentially Regulate Brain Amyloid-{beta} Peptide Clearance.. Sci Transl Med.

[41] Kuhnke D, Jedlitschky G, Grube M, Krohn M, Jucker M (2007). MDR1-P-Glycoprotein (ABCB1) Mediates Transport of Alzheimer’s amyloid-beta peptides–implications for the mechanisms of Abeta clearance at the blood-brain barrier.. Brain Pathol.

[42] Mandrekar S, Jiang Q, Lee CY, Koenigsknecht-Talboo J, Holtzman DM (2009). Microglia mediate the clearance of soluble Abeta through fluid phase macropinocytosis.. J Neurosci.

[43] Kandimalla KK, Scott OG, Fulzele S, Davidson MW, Poduslo JF (2009). Mechanism of neuronal versus endothelial cell uptake of Alzheimer’s disease amyloid beta protein.. PLoS One.

[44] Sullivan PM, Mace BE, Estrada JC, Schmechel DE, Alberts MJ (2008). Human apolipoprotein E4 targeted replacement mice show increased prevalence of intracerebral hemorrhage associated with vascular amyloid deposition.. J Stroke Cerebrovasc Dis.

[45] Filippini N, Ebmeier KP, MacIntosh BJ, Trachtenberg AJ, Frisoni GB (2011). Differential effects of the APOE genotype on brain function across the lifespan.. Neuroimage.

[46] Kok E, Haikonen S, Luoto T, Huhtala H, Goebeler S (2009). Apolipoprotein E-dependent accumulation of Alzheimer disease-related lesions begins in middle age.. Ann Neurol.

[47] Bell RD, Sagare AP, Friedman AE, Bedi GS, Holtzman DM (2007). Transport pathways for clearance of human Alzheimer’s amyloid beta-peptide and apolipoproteins E and J in the mouse central nervous system.. J Cereb Blood Flow Metab.

[48] Deane R, Zlokovic BV (2007). Role of the blood-brain barrier in the pathogenesis of Alzheimer’s disease.. Curr Alzheimer Res.

[49] Hawkes CA, McLaurin J (2009). Selective targeting of perivascular macrophages for clearance of beta-amyloid in cerebral amyloid angiopathy.. Proc Natl Acad Sci U S A.

[50] Wu Z, Guo H, Chow N, Sallstrom J, Bell RD (2005). Role of the MEOX2 homeobox gene in neurovascular dysfunction in Alzheimer disease.. Nat Med.

[51] Bell RD, Deane R, Chow N, Long X, Sagare A (2009). SRF and myocardin regulate LRP-mediated amyloid-beta clearance in brain vascular cells.. Nat Cell Biol.

[52] Sullivan PM, Han B, Liu F, Mace BE, Ervin JF (2011). Reduced levels of human apoE4 protein in an animal model of cognitive impairment.. Neurobiol Aging.

[53] Salloway S, Sperling R, Gilman S, Fox NC, Blennow K (2009). A phase 2 multiple ascending dose trial of bapineuzumab in mild to moderate Alzheimer disease.. Neurology.

[54] Boche D, Zotova E, Weller RO, Love S, Neal JW (2008). Consequence of Abeta immunization on the vasculature of human Alzheimer’s disease brain.. Brain.

[55] Sullivan PM, Mezdour H, Aratani Y, Knouff C, Najib J (1997). Targeted replacement of the mouse apolipoprotein E gene with the common human APOE3 allele enhances diet-induced hypercholesterolemia and atherosclerosis.. J Biol Chem.

