# Supplementary figures and images for: Disruption of Arterial Perivascular Drainage of Amyloid-β from the Brains of Mice Expressing the Human APOE ε4 Allele
Source: PLoS One. 2012 Jul 25;7(7):e41636. doi: 10.1371/journal.pone.0041636 (PMC3404985; doi:10.1371/journal.pone.0041636)

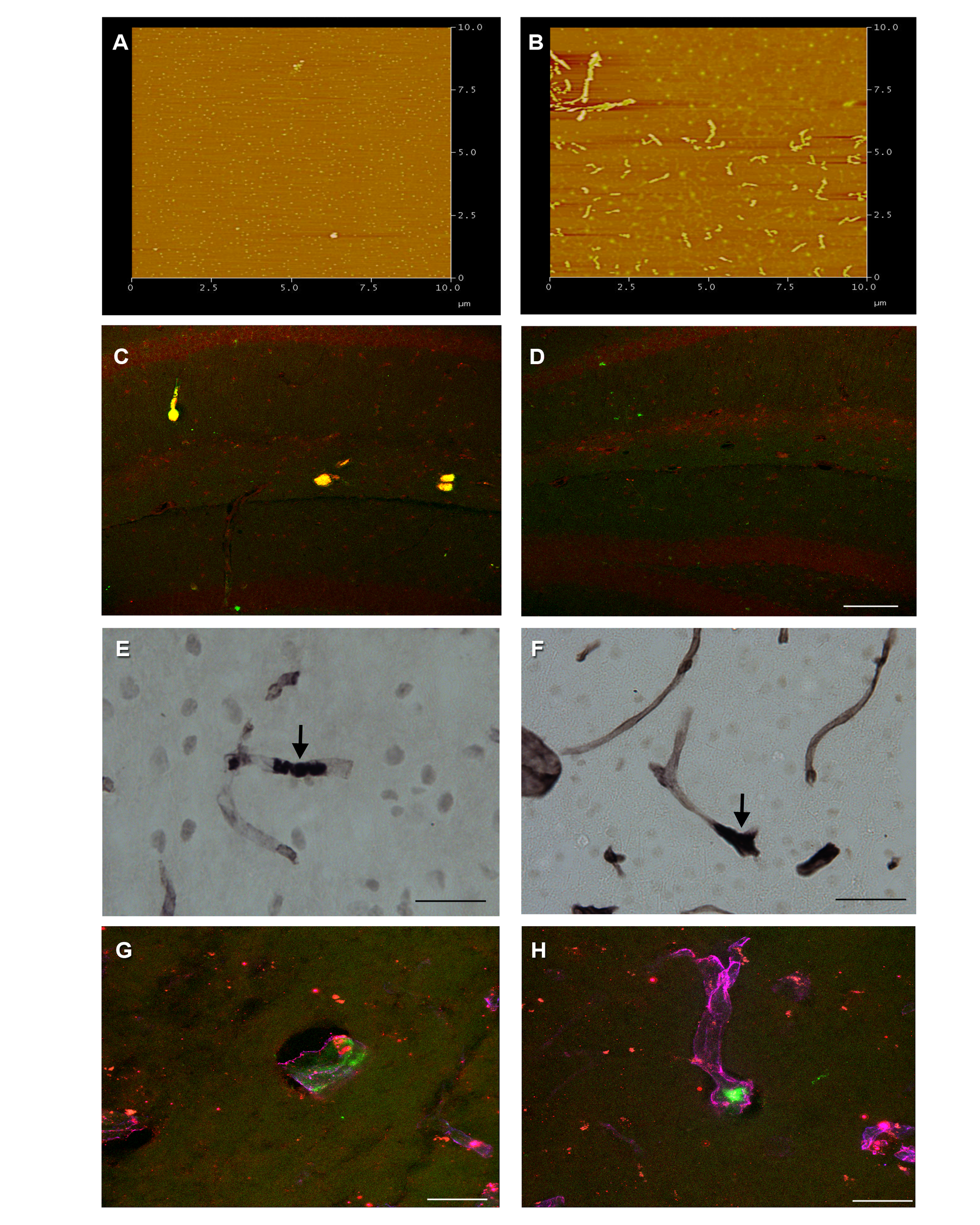

Supplement: Figure S1 — Soluble Aß injected into the hippocampus of TRE4 mice drains along basement membranes and co-localizes with apoE. a and b: HiLyte Fluor™ 488-labeled human Aβ40 used for intra-hippocampal injections was confirmed by atomic force microscopy to be oligomeric (a), compared to the fibrillar Aβ that resulted following 24 hrs incubation at 37°C (b). c and d: Brain tissue sections from 16-month old TRE4 mice were processed for Aβ immunoreactivity using a pan anti-Aβ antibody that recognized both mouse and human Aβ. The anti-Aβ antibody localized predominantly to the human Aβ (green) that had been injected into the ipsilateral hippocampus, while little to no mouse Aβ (red) was detected in the vessels in either the ipsi- (c) or contralateral hippocampus (d). e and f: Laminin staining in the vessel wall of capillaries in the 16-month old TRE4 mice (arrows) matched that of the Aβ deposits in the same animals. g and h: Double labeling immunocytochemistry with antibodies against laminin (blue) and apoE (red) in 16-month old TRE3 (g) and TRE4 (h) mice injected with human Aβ40 (green), showed localization of apoE with the basement membrane in cortical and leptomeningeal arteries. Scale bars: a and b = 10 µm; c and d = 50 µm; e–h = 25 µm. (TIF) [file pone.0041636.s001.tif]
